# Role of Pel and Psl polysaccharides in the response of *Pseudomonas aeruginosa* to environmental challenges: oxidative stress agents (UVA, H_2_O_2_, sodium hypochlorite) and its competitor *Staphylococcus aureus*

**DOI:** 10.1099/mic.0.001301

**Published:** 2023-02-09

**Authors:** Romina Grossich, Martín Lemos Vilches, Cristina S. Costa, Magdalena Pezzoni

**Affiliations:** ^1^​ Departamento de Radiobiología, Comision Nacional de Energía Atómica, San Martín, Buenos Aires, Argentina

**Keywords:** H_2_O_2_, Pel/Psl, *Pseudomonas aeruginosa*, sodium hypochlorite, UVA

## Abstract

*
Pseudomonas aeruginosa* is a versatile bacterium capable of adapting to a wide range of stress factors, including solar UVA radiation (400–315 nm). High UVA doses produce lethal effects due to the action of reactive oxygen species. Sublethal UVA doses also induces oxidative damage, but, in addition, it triggers a variety of adaptive responses, including the overexpression of *pelA* and *pslA* genes in *P. aeruginosa *. These genes encode the synthesis of Pel and Psl, which are essential polysaccharides in biofilm formation. The present study analysed the role of Pel and Psl in the adaptive responses generated by exposure to low UVA doses, and their importance in the response to lethal doses of UVA, hydrogen peroxide (H_2_O_2_), and sodium hypochlorite, in both planktonic cells and submerged and air–liquid interface (ALI) biofilms. It also studied the roles of Pel and Psl in *P. aeruginosa–Staphylococcus aureus* interaction. The results demonstrate that the capacity of sublethal UVA exposure to increase cell hydrophobicity and cell attachment and generate cross-protection phenomena in *P. aeruginosa* depends on the presence of Pel and Psl. The study also shows that Pel and Psl have a key role in the tolerance to lethal doses of UVA radiation, sodium hypochlorite and H_2_O_2_, in both biofilms and planktonic cells. Finally, co-culture assays showed total inhibition of *S. aureus* growth in presence of *P. aeruginosa *. This phenomenon depends, at least in part, on the simultaneous presence of Pel and Psl in planktonic cells and biofilms, suggesting a relevant role of these polysaccharides in the interaction between these species.

## Introduction

*Pseudomonas aeruginosa* is a micro-organism able to prosper in diverse environments, including soil, water, insects, plants and animals [1]. In humans, it is an opportunistic pathogen capable of causing acute and chronic infections. *P. aeruginosa* is also well known for causing chronic infections in people with the genetic disease cystic fibrosis (CF) [2]. Its ubiquity is partly due to its ability to form strong biofilms when it adheres to a surface suitable for the development of a biofilm matrix. During their development, *P. aeruginosa* cells secrete macromolecules known as extracellular polymeric substances (EPS), which play important roles in biofilm formation and structure, and in protecting cells from environmental injuries [3–7]. It has been widely demonstrated that biofilms are less susceptible than planktonic cells to detergents, radiation, antimicrobials and the host immune system [7–9].

The dominant EPS in the extracellular matrix of *P. aeruginosa* biofilms are the polysaccharides alginate, Pel and Psl, whose chemical structures and biosynthetic mechanisms are quite different [10, 11]. Alginate is a branching polysaccharide composed of a linear copolymer of β-d-mannuronic and α−1-guluronic acids [12, 13]. It is the predominant extracellular polysaccharide of the matrix only in mucoid variants, which are produced in CF environments [2, 14, 15]. Previous studies have shown that alginate present in biofilms provides protection against antibiotics and the immune response, and has an important role in the antioxidative defence as a reactive oxygen species (ROS) scavenger [16–19]. Non-mucoid strains, the more frequent *P. aeruginosa* phenotype, produce mainly Pel and Psl during biofilm formation [20]. Pel, a positively charged polysaccharide, is a partially de-N-acetylated linear polymer of α−1,4-N-acetylgalactosamine comprised predominantly of dimeric repeats of galactosamine and N-acetylgalactosamine [21, 22]. It is relevant in surface attachment, especially in the formation of a pellicle at the air–liquid interface (ALI). It also plays an important role in the latest maturity stages of the biofilm [23]. Moreover, it has been shown to aid in cell–cell adherence, DNA crosslinking, and protection against aminoglycoside antibiotics [21, 24], and to have an important role on the organization and interaction in mixed biofilms with other species [25]. Psl is a mannose-, glucose- and rhamnose-rich polysaccharide involved in the initial attachment to biotic or abiotic surfaces and in cell–cell interaction [26, 27]. Like Pel, Psl has a relevant role in the interaction with other species in mixed biofilms [25]. Murakami *et al*. [28] recently reported that Psl has a critical function in antibiotic tolerance, and found that stationary phase cultures of the *psl* mutant have lower tolerance to hydrogen peroxide (H_2_O_2_), suggesting that Psl is an important factor in the defence against oxidative stress. Finally, Pel and Psl provide an adaptive advantage to *P. aeruginosa* in its interaction with *Staphylococcus epidermidis* [29]. On the other hand, studies related to biofilms formed by *P. aeruginosa* and *Staphylococcus aureus *, two micro-organisms often associated in pathogenic processes [30–33] reported that these exopolysaccharides only affect the organization and integration of each species in the biofilm [25, 34].

Solar ultraviolet-A radiation (UVA, 400–315 nm), the major fraction of ultraviolet radiation reaching the Earth’s surface, is one of the main environmental stress agents for bacteria [35–37]. Exposure of bacteria to high doses of UVA produces oxidative damage to proteins, lipids and DNA, with the consequent loss of bacterial viability [38–41]. The damage is produced by the action of the ROS singlet oxygen, H_2_O_2_, superoxide anion and hydroxyl radical, which are generated by the absorption of UVA by endogenous photosensitizers, e.g. flavoproteins, cytochromes and quinones, in the presence of oxygen [42, 43]. On the other hand, exposure of bacteria to sublethal doses of UVA has also been shown to produce effects, without significant cell death. These effects include transient inhibition of bacterial growth [44] due to a direct effect of UVA on certain tRNAs causing increased ppGpp levels [45, 46] and oxidative disturbance of bacterial membranes [47]. In *P. aeruginosa *, these effects are accompanied by adaptive phenomena able to mitigate possible subsequent lethal UVA doses, such as induction of enzymatic antioxidative systems [48], increased biofilm formation [49, 50], higher proportion of membrane unsaturated fatty acids and membrane fluidity [51]. In a recent study, we demonstrated that alginate has a key role in the resistance of *P. aeruginosa* to lethal UVA doses, and in the occurrence of adaptive responses, such as induction of biofilm formation and cross-protection phenomena [19]. We also demonstrated that the UVA-dependent increase of biofilm formation is accompanied by an increase in alginate concentration in the biofilm matrix, possibly through the ppGpp-dependent induction of genes related to alginate regulation (*algR* and *algU*) and biosynthesis (*algD* operon) [19]. Similarly, we reported that UVA is able to induce the *pelA* and *pslA* genes involved in Pel and Psl biosynthesis [50], though their roles in the response to UVA exposure are unknown.

In this work, we used derivative mutants of the *P. aeruginosa* strain PAO1 unable to produce Pel and/or Psl, to conduct a comprehensive study analysing the participation of these polysaccharides in the adaptive responses generated by exposure to low doses of UVA, and their importance in the survival to lethal doses of this radiation. We also analysed the roles of Pel and Psl in the response to toxic levels of the commonly used oxidative agents H_2_O_2_ and sodium hypochlorite, and in *P. aeruginosa–S. aureus* interaction.

## Methods

### Bacterial strains and culture conditions

Table 1 lists the strains used in this study. Bacterial cultures were routinely grown at 37 °C with shaking in complete Luria–Bertani (LB) broth. For solid medium, 15 g l^−1^ agar was added.

**Table 1.** Strains used in this study

Strain	Relevant genotype and/or phenotype	Source of reference
*** P. aeruginosa ***		
PAO1	Wild type	[75]
*PAO1*∆*pel*	*pelA*; polar mutant of the *pel* operon; markerless	[76]
*PAO1 ∆psl*	*pslBCD*; polar mutant of *psl* operon; markerless	[77]
*PAO1 ∆pel psl*	∆*pelA pslBCD*; markerless	[76]
PAO1 *wspF*	*wspF*; mutant of the *wsp* operon; markerless	[78]
*** S. aureus ***		
ATCC 29213	Wild-type	[79]

### Irradiation source

Cell suspensions were irradiated using a bench with two Philips TL-D 18W/08 tubes (>95 % UVA emission at 365 nm). Irradiance under experimental conditions was measured at the surface of the suspensions with a 9811.58 radiometer (Cole-Parmer Instruments). The UVA tubes were mounted on aluminium anodized reflectors to enhance the irradiance on the section to be irradiated. The irradiances employed in this study may be encountered normally in the environment [52].

### Growth of planktonic cells and cell attachment under exposure to sublethal UVA doses

#### Planktonic cells

The procedure is based on a previous paper [49], with modifications. Mid-exponential cultures (OD_650_ 0.3) were diluted to OD_650_ 0.05 in LB medium and divided into two 15 ml fractions, each of which was placed in a glass beaker (4.5 cm internal diameter). The beakers were placed in a multi-chamber coupled to a thermo cycler bath to maintain the temperature of the suspensions at 37 °C. The cell suspensions were stirred continuously with a magnetic bar. One of the fractions was irradiated from above at an irradiance of 25 W m^−2^ at the level of the free surface of the suspension, while the other was covered with a black plastic sheet (dark control). Cell growth and the level of oxidative damage of irradiated and control suspensions were followed by measuring the OD at 650 nm and chemiluminescence values, respectively. The OD_650_ data were fitted to a polynomial regression using the programme Origin Pro 7 (Origin Lab Corporation). The equation and parameters used to obtain the fitted curves are given in Fig. S1 (available in the online version of this article).

#### Cell attachment

Mid-exponential cultures (OD_650_ 0.3) were diluted to OD_650_ 0.1 in LB medium and 1 ml aliquots of these suspensions were placed in 24 multi-well plates. The plates (without covers) were placed in a multi-chamber coupled to a thermo cycler bath so that the temperature of the suspensions was maintained at 37 °C. UVA-treated samples were irradiated from above at an irradiance of 25 W m^−2^ at the level of the free surface of the suspensions, while control samples were covered with a black plastic sheet (dark control). After 60 or 120 min, the multi-well plates were removed and the cell mass and matrix in each well was quantified by crystal-violet staining, as described later. To evaluate the number of UVA-treated and control bacteria attached to the wells, the c.f.u. were quantified. To this purpose, the cultures were removed, and the wells were washed to remove unattached cells. The bacterial biomass was scraped with a sterile plastic spatula, recovered in 0.5 ml of saline solution, and homogenized by vigorous vortexing. Appropriate dilutions of these suspensions were plated on LB solid medium. Plates were incubated for 24 h at 37 °C and the number of colonies was expressed as c.f.u.

### Chemiluminescence assays

Photoemissive species production was followed by means of a liquid scintillation system in the out-of-coincidence mode [53]. For this purpose, 1 ml aliquots were taken during bacterial growth and quickly transferred to the scintillation system equipped with photomultipliers sensitive in the blue region up to 600–650 nm (Tri-Carb 1500; Packard Instruments). Chemiluminescence values were expressed as counts per minute (c.p.m.) per OD_650_ unit.

### Crystal-violet staining

Crystal-violet staining was employed to quantify the cell mass and matrix attached to the walls of 24 multi-well plates. To do so, the culture medium was removed, the wells were washed with distilled water to remove unattached cells and 0.1 % (w/v) aqueous crystal-violet solution was added for 30 min. Then, the crystal-violet solution was discarded, and the wells were washed with distilled water to remove residual stain. The crystal violet adhered to each well was dissolved in 2 ml of a mixture of 96 % ethanol and 30 % acetic acid (1 : 1). Absorbance at 575 nm was measured in the resulting solutions.

### Cell-hydrophobicity quantification

Cell hydrophobicity was quantified by means of a test, which evaluates microbial adhesion to hydrocarbons (MATH test) [49, 54, 55]. Briefly, control and irradiated (30 min at irradiance of 25 W m^−2^) suspensions were mixed with toluene at a volume ratio of 1 : 1, and the mixtures were incubated for 30 min at 30 °C. Hydrophobicity was calculated from the difference in the aqueous phase at OD_600_before and after incubation [49].

### Evaluation of sensitivity to sodium hypochlorite and hydrogen peroxide post-sublethal UVA exposure

For cross-protection assays, 50 µl of cell cultures grown under UVA or in the dark were diluted in 2 ml of molten LB (0.75 % agar) and layered on LB plates. Sterile filter paper discs saturated with 8 µl of 0.6 % sodium hypochlorite or 8 µl of 200 µMH_2_O_2_ were then placed on the layer. Sensitivity was recorded as the diameter of growth inhibition after 24 h growth at 37 °C.

### UVA sensitivity assays

#### Planktonic cells

Overnight cultures were washed once and suspended in saline solution (NaCl 0.1M) at an OD_650_ of 0.4. Each suspension was divided into two 15 ml fractions, each of which was placed in a glass beaker (4.5 cm internal diameter). The beakers were placed in a multi-chamber coupled to a thermo cycler bath so that the temperature of the suspensions was maintained at 20 °C. One of the fractions was irradiated from above at an irradiance of 20 W m^−2^ for 180 min (radiant exposure 216 kJ m^−2^), while the other was covered with a black plastic sheet (dark control). This irradiance was lower than the one used to generate a sublethal stress level (irradiance 25 W m^−2^) because, unlike LB medium (employed in experiments of sublethal UVA assays), saline solution does not absorb radiation at 365 nm (Fig. S2). The cell suspensions were stirred continuously with a magnetic bar. Samples were taken at regular intervals from both fractions, diluted in saline solution and plated on LB solid medium to assess cell viability by counting the number of c.f.u. Plates were incubated in the dark to prevent light-induced DNA repair and the colonies were counted after 24 h incubation at 37 °C. Survival was expressed as the fraction of the c.f.u. ml^−1^ at time 0.

#### Biofilms

Overnight cultures were diluted to an OD_650_ of 0.1 in LB medium, and submerged and ALI biofilms were developed. For submerged biofilms, 10 ml of each suspension were placed in 100 ml glass beakers, each containing a sterile glass slide (20 mm × 25 mm × 1 mm) placed horizontally on the bottom. For ALI biofilms, 5 ml of each suspension was placed in 50 ml Falcon tubes containing the slides placed vertically at the bottom so that about half of the slide was submerged. The beakers and tubes were capped with cotton plugs and incubated at 37 °C for 24 h without shaking to allow biofilm formation on the slides. The slides supporting submerged or ALI biofilms were removed, washed once with distilled water to eliminate unattached cells, and placed in small Petri dishes (diameter 5 cm) open to air and covered with 5 ml saline solution. For UVA treatment, biofilms were irradiated from above at an irradiance of 20 W m^−2^ for 120 min (radiant exposure 144 kJ m^−2^), while control biofilms were kept in the dark by covering the plates with a black plastic sheet. To evaluate the cell viability in UVA-treated and control biofilms, the c.f.u. per area unit was quantified. To do this, the slides supporting the biofilms were removed and washed by letting sterile distilled water (about 10 ml) drop down gently on them to remove unattached cells. The bacterial biomass was then scraped from the glass with a sterile plastic spatula, recovered in 0.5 ml of saline solution, and homogenized by vigorous vortexing. Appropriate dilutions of these suspensions were plated on LB solid medium. Plates were incubated in the dark immediately after irradiation and the colonies were counted after incubation for 24 h at 37 °C. Survival was expressed as a fraction of the c.f.u. cm^−2^ at time 0.

### H_2_O_2_ and sodium hypochlorite sensitivity assays

#### Planktonic cells

Sensitivity assays to H_2_O_2_ were based on a previous paper [56], with modifications. Briefly, stationary phase cells were diluted in 20 ml of LB medium until 1×10^8^ c.f.u. ml^−1^, added with H_2_O_2_ (final concentration 50 mM) and incubated at 37 °C with agitation. The same procedure was followed in the case of sodium hypochlorite, but it was added at a final concentration of 0.25 %. Appropriate dilutions of the suspensions were plated on LB solid medium before and after 10 min of treatment. The plates were incubated at 37 °C and the colonies were counted after 24 h. Survival was expressed as the fraction of c.f.u. ml^−1^ at time 0.

#### Biofilms

Slides supporting 24 h submerged or ALI biofilms obtained as described above were removed from the beakers, washed once with distilled water to eliminate unattached cells, and placed in small Petri dishes (diameter 5 cm) open to air. The samples were covered with 5 ml of saline solution, and H_2_O_2_ (final concentration 50 mM) or sodium hypochlorite (final concentration 1%) were added for 30 or 15 min, respectively. To evaluate cell survival, c.f.u. per area unit was quantified before and after treatment, as described above for UVA sensitivity assays. Survival was expressed as the fraction of the c.f.u. cm^−2^ at time 0.

### Co-cultures of *P. aeruginosa* and *S. aureus*

#### Planktonic cells

Stationary phase cultures of the *P. aeruginosa* strains and *S. aureus* were diluted with fresh LB to OD_650_ 0.1 and mixed in equal volumes (5 ml). Controls from single cultures were prepared under similar conditions. Control and mixed cultures were grown at 37 °C for 24 h with aeration, and appropriate dilutions were plated on LB solid medium. The plates were incubated at 37 °C for 24 h and the c.f.u. ml^−1^ of each species were quantified. *S. aureus* presented characteristic yellow, round, opaque and convex colonies, easily differentiated from the colonies of the *P. aeruginosa* strains. To illustrate this, representative photographs showing the colony morphology of all the strains used in this study (in single and mixed cultures) are shown in Fig. S3.

#### Biofilms

Overnight cultures of the *P. aeruginosa* strains and *S. aureus* were diluted to an OD_650_ of 0.1 in LB medium. Then, 5 ml of these suspensions were mixed at 1 : 1 ratio and used to form submerged or ALI biofilms as described above. To quantify the cell number from each species in the mixed biofilms, slides were removed and the biofilms recovered as described above. Appropriate dilutions of the suspensions were plated on LB solid medium and incubated for 24 at 37 °C, when the c.f.u. cm^−2^ of each species was quantified. Single biofilms obtained under similar conditions were quantified as control.

### Statistical analysis

All samples were analysed at least in triplicate. Data are shown as mean±se. Tests for significance between means were carried out using Student’s *t*-test with confidence levels at >95 % and one-way or two-way ANOVA and Tukey’s multiple-comparison post-test. The employed test is detailed in each figure caption. Differences between groups were considered to be significant at a *P* value of <0.05. Statistical analyses were performed with GraphPad Prism 8.0 (GraphPad Software, San Diego, CA).

## Results

### Pel and Psl protect planktonic growing cells from oxidative damage generated by UVA

**Fig. 1.**
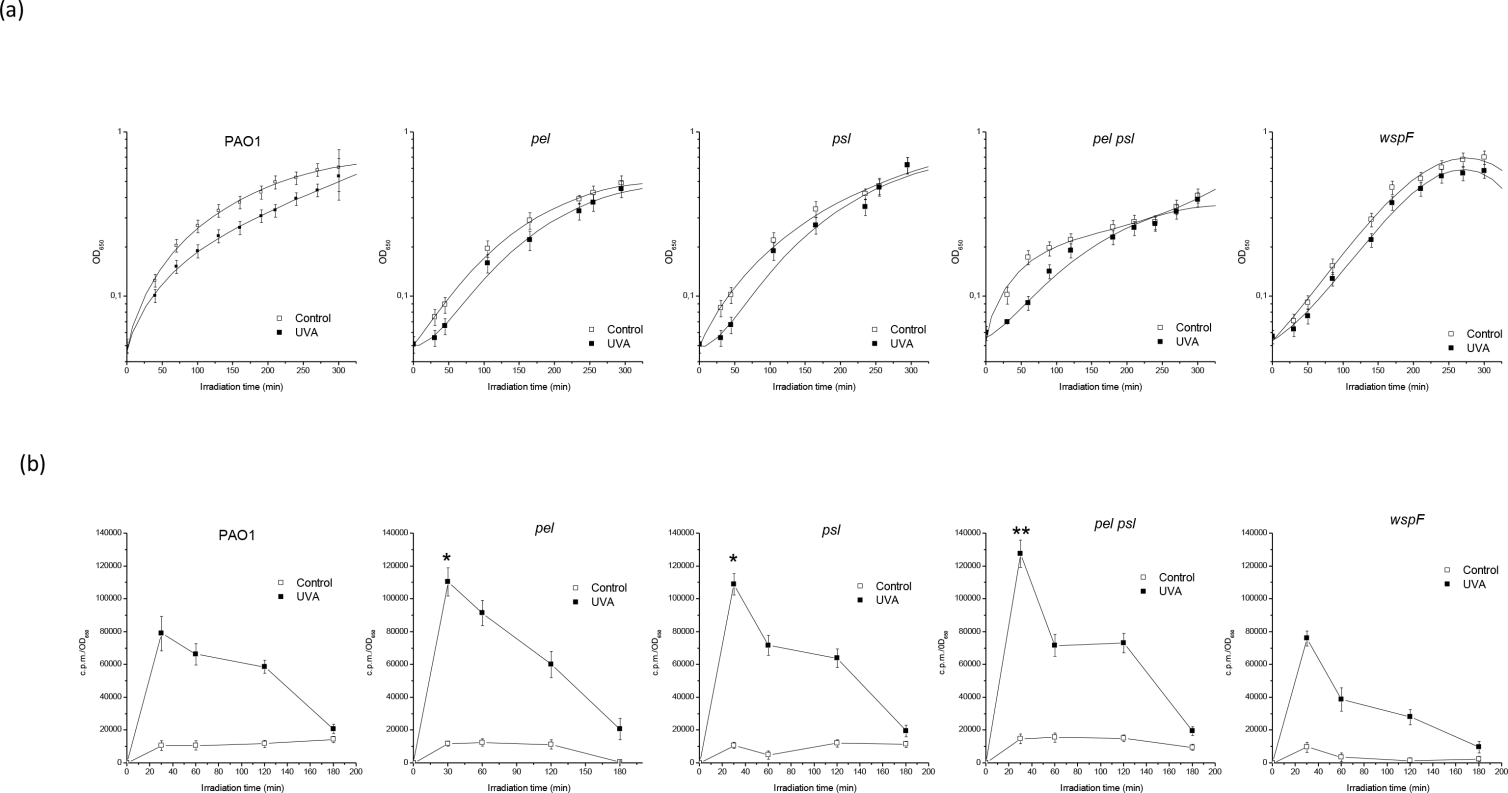
Effect of exposure to sublethal UVA doses (irradiance 25 Wm^−2^) on the growth (a) and chemiluminescence (b) of PAO1, *pel*, *psl*, *pel psl* and *wspF* strains. Control cells were grown under similar conditions but kept in the dark. Test for significance was carried out using Student’s *t*-test. *P<0.05; **P<0.005.

Growth under sublethal doses of UVA of the PAO1 strain and its derivative mutants unable to produce Pel or/and Psl (*pel*, *psl* and *pel psl* strains) was analysed. This study also included a *wspF* mutant, which presents a deletion in the *wspF* gene that results in an elevated production of the matrix protein CdrA and the secondary messenger cyclid-di-GMP, with the consequent over-production of Pel and Psl [57, 58]. Strains were grown in complete medium at 37 °C under UVA at an irradiance of 25 Wm^−2^, while control cells were maintained in the dark. This condition, utilized in previous studies [19, 51], did not alter cell viability significantly but produced some oxidative damage. Growth curves are shown in Fig. 1(a). All the strains were affected by the radiation, compared to their respective control cultures, but it was possible to observe that in the mutants deficient for Pel and/or Psl synthesis this effect was more pronounced. The results were explained taking into account the slopes of plotted lines at the first part (between 0 and 30 min approximately) of the fitted curves. The slopes of the UVA curves of PAO1, *wspF*, *pel*, *psl* and *pel psl* strains corresponded to about 82, 85, 75, 65 and 30 % of the slopes of the corresponding control curves, respectively. In order to evaluate if oxidative damage was associated to the UVA induced-growth delay, the ultraweak chemiluminescence procedure was employed [47, 59]. As shown in Fig. 1(b), all strains displayed a strong peak at the beginning of the exposure, indicating oxidative damage. The *pel* and *psl* single mutants showed significantly higher peaks than the PAO1 strain (*P*<0.05), and the peak of the *pel psl* strain was the highest (*P*<0.005). The *wspF* showed the same response as the PAO1 strain. These results suggest that oxidative damage occurs under the conditions employed, and is more evident in the absence of Pel and/or Psl. Control cells maintained in the dark did not show relevant changes in chemiluminescence values.

### Pel and Psl participate in the UVA-dependent induction of cell attachment

To analyse the role of Pel and Psl in the UVA-promoted cell attachment phenomenon [49, 50], we quantified the cell mass and matrix (measured by crystal-violet staining as absorbance at 575 nm) and the number of c.f.u. attached to multi-well plates, after 60 or 120 min exposure to UVA or in the dark.

**Fig. 2.**
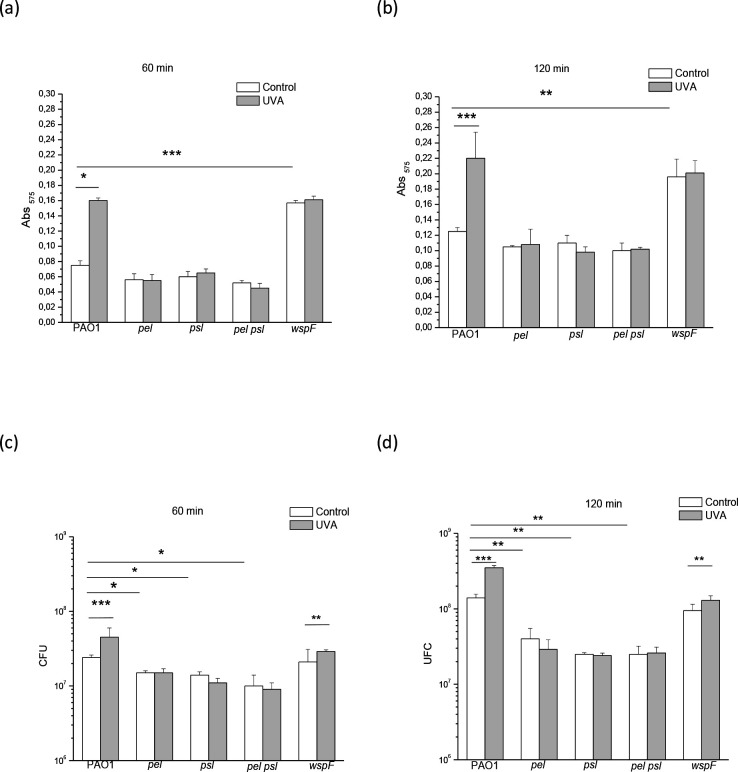
Effect of exposure to sublethal UVA doses (irradiance of 25 Wm^−2^) for 60 (a, c) or 120 (b, d) min on cell attachment of PAO1, *pel*, *psl*, *pel psl* and *wspF* strains. Cell attachment was measured by crystal-violet staining (a, b) and c.f.u. counting (c, d). Control biofilms were maintained in the dark under identical conditions. Test for significance was carried out using two-way ANOVA. *P<0.05; **P<0.005; ***P<0.0005.

As shown in Fig. 2(a, b), UVA radiation delivered for 60 and 120 min at an irradiance of 25 W m^−2^ (radiant exposure 90 and 180 kJ m^−2^) increased the cell mass and matrix of the PAO1 strain compared to the control condition (*P*<0.05 and *P*<0.0005, respectively). However, in the case of *pel*, *psl* and *pel psl* mutant strains, no significant increase in A_575_ was observed by the treatment; in the dark, the PAO1 strain and these mutants did not differ significantly in this regard. When the number of c.f.u. was evaluated (Fig. 2c, d), a significant increase was observed when the PAO1 strain was exposed to UVA (*P*<0.0005), and this effect was not observed in the *pel*, *psl* and *pel psl* mutants. Under the control condition, the c.f.u. counts in mutants deficient for Pel and/or Psl was lower (*P*<0.05 at 60 min and *P*<0.005 at 120 min) compared to the wild-type. On the other hand, the *wspF* strain showed a significant (*P*<0.0005 at 60 min and *P*<0.005 at 120 min) higher level of A_575_ in the control condition compared to the wild-type (Fig. 2a, b), probably due to its intrinsically high polysaccharide production, which did not increase by the UVA exposure. However, as in PAO1, in this strain the treatment increased the c.f.u. number (*P*<0.005) (Fig. 2 c, d).

### UVA-dependent increase in cell hydrophobicity depends on Pel and Psl

**Fig. 3.**
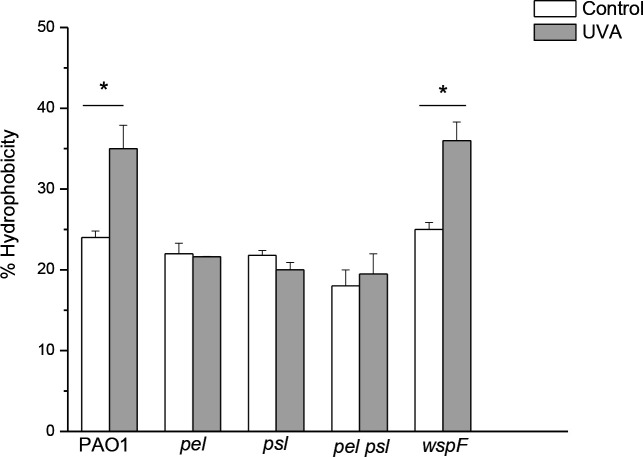
Effect of exposure to sublethal UVA doses (30 min irradiation at irradiance of 25 Wm^−2^) on cell hydrophobicity of PAO1, *pel*, *psl*, *pel psl* and *wspF* strains. Control cells were grown in the dark under identical conditions. Test for significance was carried out using two-way ANOVA. *P<0.0005.

The MATH test was assayed in the wild-type and the mutants used in this study to investigate whether Pel and Psl are involved in the increase of cell hydrophobicity by UVA exposure [49]. As shown in Fig. 3, when the planktonic cells were grown under UVA, cell hydrophobicity did not significantly increase in the mutant strains *pel*, *psl* and *pel psl*. In contrast, a significant increase in this parameter was observed in the wild-type as well as in the *wspF* strain (*P*<0.0005).

### Pel and Psl are essential in cross-protection phenomena against H_2_O_2_ and sodium hypochlorite

PAO1 and the mutant strains w*spF, pel*, *psl* and *pel psl* were grown under sublethal UVA doses or in the dark, and then exposed to H_2_O_2_ and sodium hypochlorite in order to analyse the role of Pel and Psl under these conditions.

**Fig. 4.**
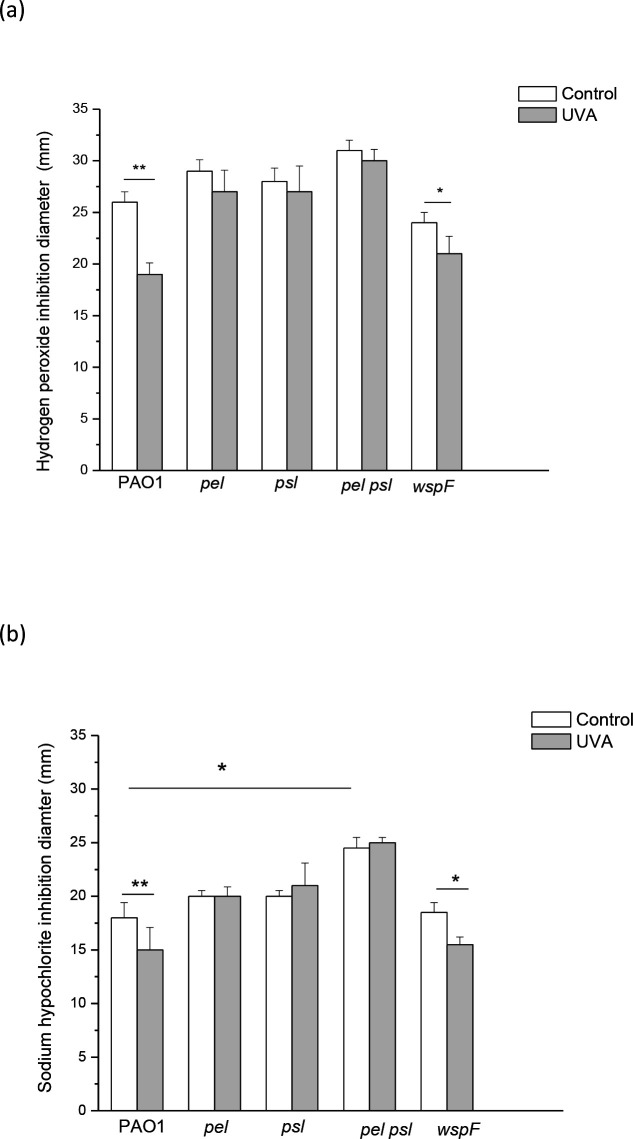
Effect of exposure to sublethal UVA doses (120 min irradiation at irradiance of 25 Wm^−2^) on sensitivity of PAO1, *pel*, *psl*, *pel psl* and *wspF* strains to (a) H_2_O_2_ and (b) sodium hypochlorite .Control cells were grown in the dark under similar conditions. Test for significance was carried out using two-way ANOVA. *P<0.005; **P<0.0005.

The analysis of cross-protection against to H_2_O_2_ showed a significant increase in the viability of pre-exposed PAO1 and *wspF* cells (*P*<0.0005 and *P*<0.005, respectively) (Fig. 4a). In contrast, no significant differences were observed in the *pel*, *psl* and *pel psl* mutants exposed to this treatment. Similar results were observed for sensitivity to sodium hypochlorite, but, in this case, it could be observed under the control condition higher sensitivity of the double mutant *pel psl* compared to the wild-type (*P*<0.005) (Fig. 4b).

### Pel and Psl protect *P. aeruginosa* planktonic cells and biofilms from lethal UVA doses

**Fig. 5.**
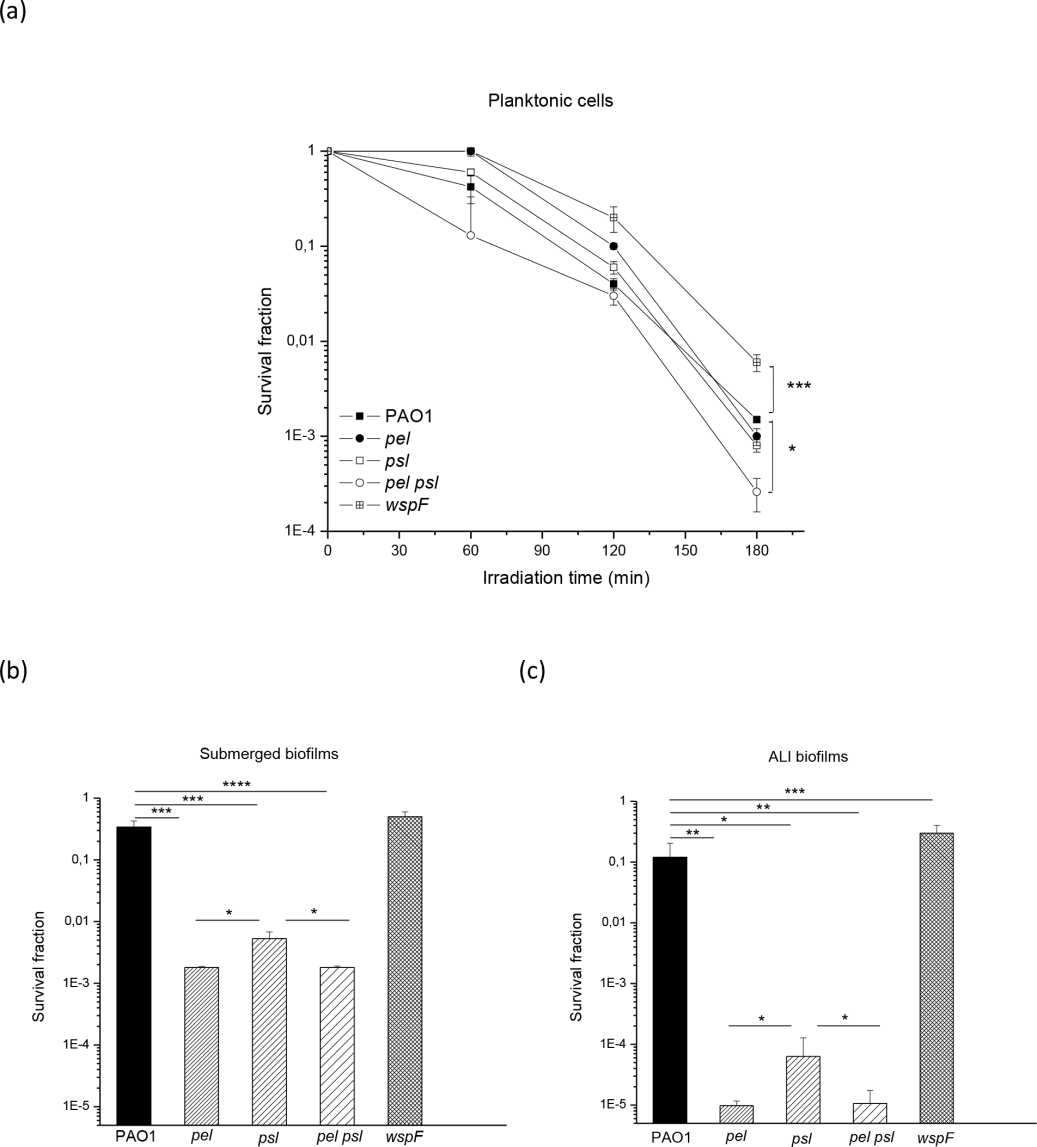
Survival of PAO1, *pel*, *psl*, *pel psl* and *wspF* strains to lethal doses of UVA radiation. Planktonic cells were exposed at an irradiance of 20 Wm^−2^ for 180 min (a), while submerged (b) and ALI (c) biofilms were exposed at the same irradiance for 120 min. Upon exposure, viability was assayed using the c.f.u. count method. Test for significance was carried out using one-way ANOVA. *P<0.05; **P<0.005;^***^ *P*<0.0005.

The next step was to study the function of Pel and Psl when lethal doses of oxidative agents were applied to planktonic cells and biofilms, beginning with UVA. Cell suspensions of the wild-type PAO1 and the mutants *wspF*, *pel*, *psl* and *pel psl* were irradiated with lethal UVA doses and their survival was determined. As shown in Fig. 5(a), the presence of at least one polysaccharide conferred protection, since the lack of Pel or Psl did not decrease the survival fraction significantly compared to the wild-type. However, survival was considerably lower in the double mutant *pel psl* strain than in the PAO1 strain and the single mutants *pel* and *psl* (*P*<0.05). Resistance to UVA was significantly higher in the *wspF* strain than in the wild-type and the single mutants *pel* and *psl* (*P*<0.0005).

This response was then analysed in submerged and ALI static 24 h biofilms. When submerged biofilms were irradiated (Fig. 5b), it was observed that the *pel, psl* and *pel psl* mutant strains were significantly more sensitive than PAO1 and the *wspF* strain (*P*<0.0005, significances not shown in the case of *wspF*). The same tendency was observed in ALI biofilms (Fig. 5c). In both submerged and ALI biofilms, *pel* and *pel psl* mutants behaved similarly and were more sensitive to UVA than the *psl* strain (*P*<0.05). Only in the case of ALI biofilms, the *wspF* strain survival was significantly higher than wild-type survival (*P*<0.0005).

### Pel and Psl protect *P. aeruginosa* planktonic cells and biofilms against lethal doses of H_2_O_2_ and sodium hypochlorite

**Fig. 6.**
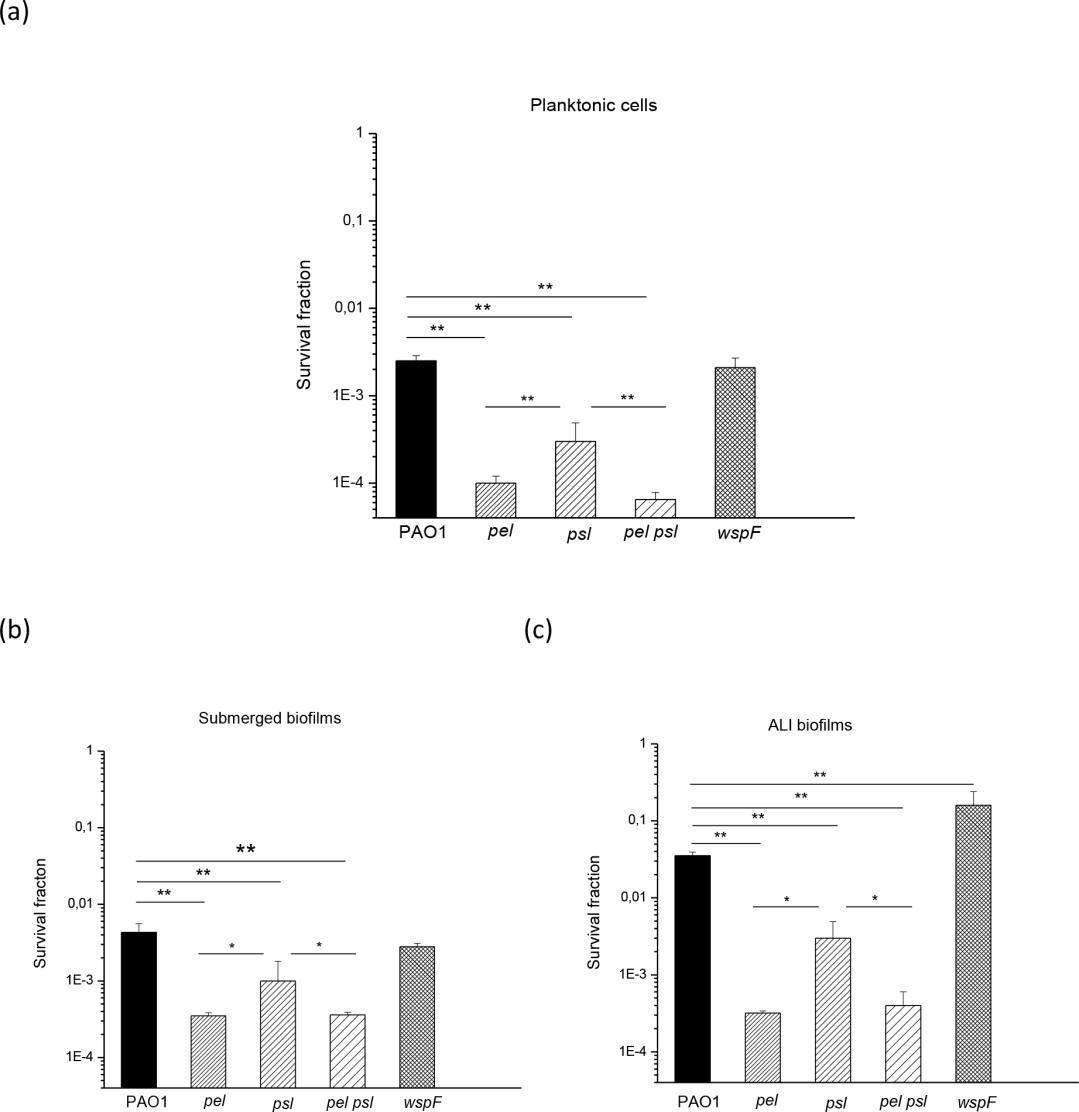
Survival of PAO1, *pel*, *psl*, *pel psl* and *wspF* strains exposed to lethal doses of H_2_O_2_. Planktonic cells were exposed to 50 mM H_2_O_2_ for 10 min (a), while submerged (b) and ALI (c) biofilms were exposed at the same H_2_O_2_concentration for 30 min. Upon exposure, viability was assayed using the c.f.u. count method. Test for significance was carried out using one-way ANOVA. *P<0.05; **P<0.0005.

**Fig. 7.**
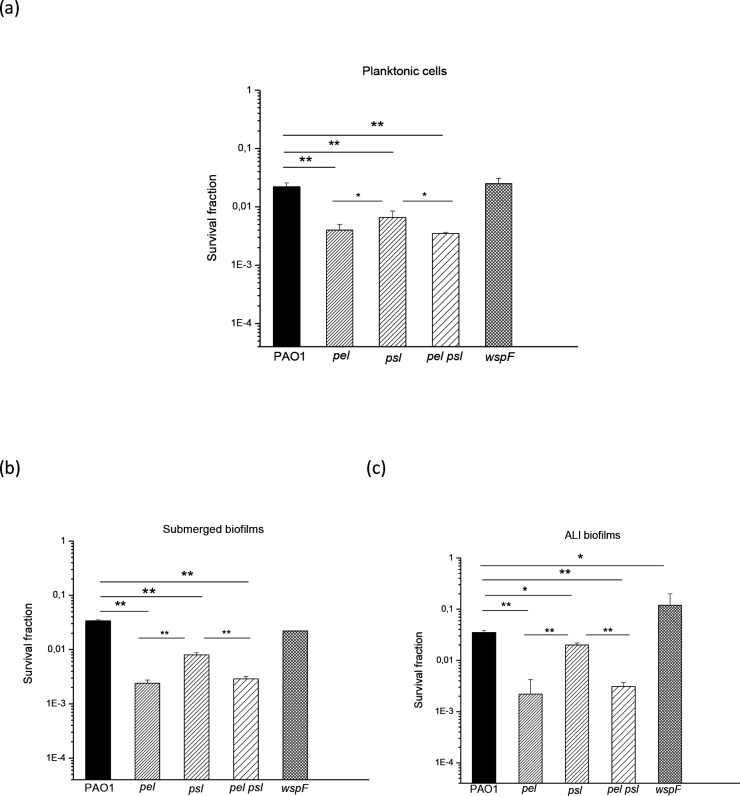
Survival of PAO1, *pel*, *psl*, *pel psl* and *wspF* strains exposed to lethal doses of sodium hypochlorite. Planktonic cells were exposed to 0.25 % sodium hypochlorite for 10 min (a), while submerged (b) and ALI (c) biofilms were exposed to 1 % sodium hypochlorite for 15 min. Upon exposure, viability was assayed using the c.f.u. count method. Test for significance was carried out using one-way ANOVA. *P<0.005; **P<0.0005.

The role of Pel and Psl in the effect of H_2_O_2_ and sodium hypochlorite was also analysed. A similar pattern was observed in both planktonic cells and biofilms. As shown in Figs 6 and 7, *pel*, *psl* and *pel psl* mutant strains were significantly more sensitive than PAO1 (*P*<0.0005, except for PAO1 vs *psl* ALI in Fig. 7(c), *P*<0.005) and the *wspF* strain (significances not shown). The mutants *pel* and *pel psl* were always significantly more sensitive compared to the *psl* strain. The *wspF* strain responded like the wild-type, except in ALI biofilms (Figs 6c and 7c), where this strain was significantly more resistant than the wild-type (*P*<0.0005 or *P*<0.005, in H_2_O_2_ or hypochlorite tests, respectively).

### *P. aeruginosa* Pel and Psl inhibit *S. aureus* growth in mixed planktonic cultures and biofilms

**Fig. 8.**
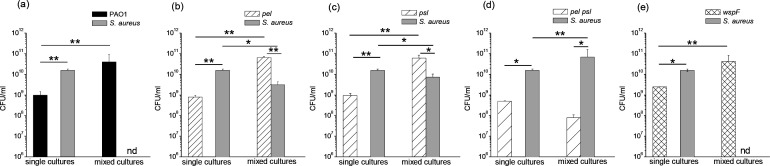
Effect of Pel and Psl polysaccharides on the growth of *S. aureus* in mixed planktonic cultures. Diluted cultures of *P. aeruginosa* (PAO1, *pel*, *psl*, *pel psl* or *wspF* strains) and *S. aureus* were mixed at 1 : 1 ratio. The mixtures were incubated at 37 °C for 24 h, and the c.f.u. ml^−1^ of each species were quantified. Controls from single cultures were quantified under similar conditions. Test for significance was carried out using one-way ANOVA. *P<0.005; **P<0.0005. nd, not detected.

We studied the role of Pel and Psl in the competition between *P. aeuginosa* and *S. aureus *. Firstly, we compared the behaviour of single and mixed planktonic cultures, by combining the PAO1 strain or the mutants employed in this study with a representative strain of S. *aureus *, as explained in Methods (Fig. 8). As shown in Fig. 8(a, e), in mixed cultures of *S. aureus* with PAO1 or the *wspF* strain, only *P. aeruginosa* cells were obtained after 24 h. In contrast, when PAO1 was replaced by *pel*, *psl* or *pel psl* strains (Fig. 8b–d), *S. aureus* recovered its growth capacity, mainly when both polysaccharides were absent (single vs mixed cultures *P*<0.005, *P*<0.005, and *P*<0.0005, respectively). In addition, all *P. aeruginosa* strains increased their cell counts after 24 h when they were grown in the presence of *S. aureus* compared to their corresponding single cultures (Fig. 8a, b, c, e; *P*<0.0005), except in the case of the *pel psl* mutant (Fig. 8d). In single cultures, *S. aureus* presented significant higher c.f.u. values compared to all the *P. aeruginosa* strains (Fig. 8a–e), while this trend is reversed in mixed cultures except for the *pel psl* strain (Fig. 8b–d).

**Fig. 9.**
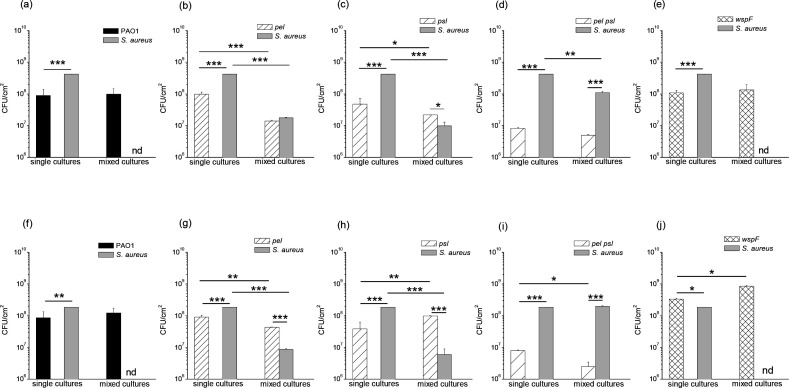
Effect of Pel and Psl polysaccharides on the growth of *S *. *aureus* in mixed biofilms. Diluted cultures of *P. aeruginosa* (PAO1, *pel*, *psl*, *pel psl* or *wspF* strains) and *S. aureus* were mixed at a 1 : 1 ratio; these mixtures were used to form submerged (a–e) or ALI (f–j) biofilms. After 24 h incubation at 37 °C, the c.f.u. cm^−2^ of each species was quantified. Single biofilms obtained under similar conditions were quantified as control. Test for significance was carried out using one-way ANOVA. *P<0.05; **P<0.005; ***P<0.0005. nd, not detected.

Submerged and ALI static biofilms were also analysed in this regard (Fig. 9). In both cases, the presence of PAO1 or *wspF* strains inhibited the development of *S. aureus* (Fig. 9a, e, f, j). In contrast, *S. aureus* was able to proliferate when it coexisted with *pel* or *psl* mutants (Fig. 9b, c, g, h; *P*<0.0005 in single vs mixed cultures), and achieved higher values in mixed biofilms when co-cultured with the double mutant *pel psl* (Fig. 9d, i; *P*<0.005 in submerged or no significant difference in ALI, single vs mixed cultures). *S. aureus* single biofilms presented significant higher c.f.u. values compared to those of *P. aeruginosa* strains (Fig. 9a–e, f–i), except in the case of ALI biofilms of the *wspF* mutants (Fig. 9j). In contrast, in mixed biofilms, the c.f.u. counts of *P. aeruginosa* strains increased significantly with respect to *S. aureus* (Fig. 9c, g, h) except in the case of the presence of *pel psl* (Fig. 9d, i) and *pel* strain, only in submerged biofilms (Fig. 9b).

## Discussion

In a series of studies conducted with *P. aeruginosa* to understand the adaptive responses triggered by UVA radiation, we demonstrated that the exposure to sublethal doses induces biofilm formation [49] by a mechanism involving activation of the transcriptional regulators ppGpp and the QS system [50]. The results presented in this study demonstrate that Pel and Psl polysaccharides play a key role in the phenomenon of UVA-induced cell attachment, the first stage of biofilm formation. Pel and Psl have been described as the predominant exopolysaccharides in *P. aeruginosa* biofilms, being critical for initial cell attachment and in the maintenance of cell–cell interactions [21, 24]. Although the regulation of *pel* and *psl* operons is not yet fully understood, Sakuragi and Kolter [60] demonstrated that the QS regulatory system is involved in their expression. Later, we demonstrated that UVA promotes transcription of regulatory QS genes (*rhlI*, *rhlR*, *lasI, lasR*) and genes encoding enzymes related to Pel and Psl synthesis (*pelA*, *pslA*) [50]. In turn, the key transcriptional regulator ppGpp, whose levels in *P. aeruginosa* increase by UVA exposure [37], governs UVA-dependent QS induction [50]. Taking all this information into account, it could be hypothesized that one of the mechanisms involved in UVA-promoted biofilm formation depends on the ppGpp-dependent QS activation of *pel* and *psl* operons. A similar pathway was recently proposed for alginate [19].

Several authors have demonstrated that Pel and Psl contribute to the surface hydrophobicity of the cell [61–63], a key factor involved in the initial attachment of bacteria to surfaces [64–66]. It has been demonstrated that in the process of induction of biofilm formation by exposure to low UVA doses, cell surface hydrophobicity and swimming motility are increased, with the consequent enhancement of cell adhesion [49]. The results presented in this study show that the increase in UVA-dependent hydrophobicity depends on the presence of Pel and Psl. Under oxidative conditions, changes in cell hydrophobicity were observed by alterations in lipopolysaccharide composition, resulting in increased cell adhesion [67]. Further studies are needed to gain greater knowledge of the changes in cell membranes (exopolysaccharides, lipopolysaccharides) produced by UVA exposure related to changes in cell hydrophobicity, and in consequence, in cell adhesion to surfaces.

In addition to biofilm formation, another adaptive response triggered by UVA exposure in *P. aeruginosa* involves protection against subsequent lethal doses of the oxidant agents sodium hypochlorite and hydrogen peroxide [19, 48]. It has been demonstrated that these cross-protection phenomena obey to increased activity of catalases KatA and KatB [48] and higher alginate levels [19]. The current study shows that Pel and Psl are also involved in this adaptive response. Their involvement in the defence against oxidative stress was confirmed by the response of *pel* and *psl* mutants exposed to lethal doses of UVA, H_2_O_2_ or sodium hypochlorite. Although the lack of either polysaccharide does not significantly decrease the survival of bacteria exposed to UVA, the absence of both increases lethality significantly. Consequently, the overproduction of Pel and Psl in the *wspF* strain improves survival. Note that because the cells are washed before UVA exposure, they lose the fraction of extracellular polysaccharides, so the effect should be due to the Pel and/or Psl attached to the cells. In addition, Pel, and to a lesser extent, Psl, also have a protective effect against H_2_O_2_ and sodium hypochlorite in planktonic cells. Although the cells were not washed in this case, they were diluted about 50-fold, so the effect is probably mainly due to the fraction of Pel and/or Psl attached to the cells, as described for UVA. It has been reported that both polysaccharides are present in at least two forms: a high molecular weight cell-associated component, and a relatively smaller soluble fraction that can be isolated from the cell-free culture supernatant [21, 22, 27]. The results on planktonic cells presented in this paper (survival tests, adaptive phenomena induced by UVA) are valuable because the participation of Pel and Psl in different cellular responses has been described mainly in biofilms, where both molecules are trapped in the matrix [28, 63, 68].

It is demonstrated that in biofilms both polysaccharides play a significant role in survival against UVA radiation. The fact that the *pel* mutant behaved similarly to the double mutant *pel psl* demonstrates that Pel has a dominant role. A similar response is observed when biofilms are exposed to lethal doses of H_2_O_2_ and sodium hypochlorite. It has been reported in biofilms that in the absence of Psl, Pel production increases in the periphery and in pellicles, suggesting that Pel could compensate the lack of Psl under a mechanism that is still unknown [11, 21]. Thus, in *psl* mutants, higher levels of Pel might counteract the lack of Psl, generating greater protection against stress agents than observed in the *pel* mutant. As suggested by Colvin *et al*. [68], Pel and Psl provide redundant functions in order to preserve the capacity to produce a biofilm when exopolysaccharide genes are subjected to mutation.

Given the oxidative nature of damage by UVA, H_2_O_2_ and sodium hypochlorite, the survival data presented in this study suggest an antioxidative role for Pel and Psl. Chemiluminescence assays provide evidence in this regard. During growth under sublethal UVA doses, a more pronounced growth delay is observed in mutants impaired for Pel and/or Psl production, and this behaviour is accompanied by higher chemiluminescence peaks. These peaks, attributed to photon emission by excited carbonyl groups and singlet O_2_ dimers arising from the decomposition of membrane lipid peroxides are indicators of oxidative damage [59]. Different hypotheses could explain an antioxidative role for Pel and Psl: (i) Psl could act as a barrier reducing ROS penetration across bacterial membranes, conferring advantages against different antimicrobials [69]; (ii) Several studies show a relevant role for Pel against different oxidative agents and aminoglycosides [24, 69, 70], suggesting that Pel acts like Psl in this regard; (iii) Pel and/or Psl could influence biofilm structure, making the cells less susceptible to oxidant agents; (iv) In the particular case of UVA, Pel and/or Psl could act as shields, absorbing or deviating the radiation and preventing photons from reaching cellular targets [71].

*P. aeruginosa* produces several molecules to compete with other micro-organisms for space and nutrients, which makes it highly successful. For example, the ability of *P. aeruginosa* to compete with *Staphylococcus* for the niche has been linked to its ability to sense it by shedding peptidoglycan [72], and to secrete numerous products including phenazines [73], extracellular matrix products [29] and proteases [74]. In particular, the species *S. aureus* is often associated with *P. aeruginosa* in pathogenic processes such as wounds or CF, constituting complex interactions with impact on temporal colonization, pathogenicity and inflammatory responses [30–33]. Under the conditions employed in this study, that is, both strains put together to grow as free cells or biofilms, the presence of the wild-type *P. aeruginosa* completely inhibits *S. aureus *. Pel and Psl seem to be critical in this association because the absence of either one allows the growth of *S. aureus* at a low level, achieving values similar to that obtained in single cultures when both polysaccharides are absent (Figs 8 and 9). A competitive advantage for *P. aeruginosa* dependent on Pel and Psl was also observed by Qin *et al*. [29], demonstrating that these polysaccharides disrupt previously formed *S. epidermidis* biofilms. On the other hand, a study related to the interaction between *P. aeruginosa* and *S. aureus* in mixed biofilms reported that these exopolysaccharides only affect the spatial organization and integration of each species [25]. Billings *et al*. [34] showed that *S. aureus* is incorporated in the air–liquid interface of a Psl-producing *P. aeruginosa* biofilm. Discrepancies between different studies on biofilms may occur because the species interactions determining biofilm structure depends on the strains used and/or the protocol employed for biofilm formation. In the case of planktonic cells, to the best of our knowledge, this is the first report about the role of these polysaccharides in the *P. aeruginosa–S. aureus* association. Further work is required to gain deeper understanding of the role of Pel and Psl in this association, for example analysing whether these polysaccharides are toxic to another bacteria or simply act by improving *P. aeruginosa* fitness.

In summary, this work demonstrates the important role of Pel and Psl in adaptive phenomena related to sublethal UVA exposure, such as increased cell attachment and cross-protection phenomena. In addition, it shows the antioxidative function of these polysaccharides, protecting *P. aeruginosa *-free cells and biofilms from lethal doses of the oxidative agents UVA, H_2_O_2_ and sodium hypochlorite. These results complement a previous study demonstrating that alginate, the other matrix polysaccharide, also has important functions in the response to oxidative agents, even in non-mucoid strains, where it is only present in small amounts [19]. It also reports the pivotal role of Pel and Psl in the *P. aeruginosa–S. aureus* interaction. *P. aeruginosa* is a much studied micro-organism because of its versatility, ubiquity and unique characteristics as an opportunistic pathogen. In this regard, it is important to understand the mechanisms involved in its responses to stress agents in nature or as a pathogen in free-living cells and biofilms.

## Supplementary Data

Supplementary material 1

## References

[1] Hardalo C, Edberg SC (1997). *Pseudomonas aeruginosa*: assessment of risk from drinking water. Crit Rev Microbiol.

[2] Govan JR, Deretic V (1996). Microbial pathogenesis in cystic fibrosis: mucoid *Pseudomonas aeruginosa* and *Burkholderia cepacia*. Microbiol Rev.

[3] Flemming HC, Wingender J (2010). The biofilm matrix. Nat Rev Microbiol.

[4] Stoodley P, Sauer K, Davies DG, Costerton JW (2002). Biofilms as complex differentiated communities. Annu Rev Microbiol.

[5] Ophir T, Gutnick DL (1994). A role for exopolysaccharides in the protection of microorganisms from desiccation. Appl Environ Microbiol.

[6] Stewart PS, Costerton JW (2001). Antibiotic resistance of bacteria in biofilms. Lancet.

[7] Harrison JJ, Turner RJ, Ceri H (2005). Persister cells, the biofilm matrix and tolerance to metal cations in biofilm and planktonic *Pseudomonas aeruginosa*. Environ Microbiol.

[8] Drenkard E (2003). Antimicrobial resistance of *Pseudomonas aeruginosa* biofilms. Microbes Infect.

[9] Pezzoni M, Pizarro RA, Costa CS (2014). Protective role of extracellular catalase (KatA) against UVA radiation in *Pseudomonas aeruginosa* biofilms. J Photochem Photobiol B.

[10] Franklin MJ, Nivens DE, Weadge JT, Howell PL (2011). Biosynthesis of the *Pseudomonas aeruginosa* extracellular Polysaccharides, Alginate, Pel, and Psl. Front Microbiol.

[11] Ghafoor A, Hay ID, Rehm BHA (2011). Role of exopolysaccharides in *Pseudomonas aeruginosa* biofilm formation and architecture. Appl Environ Microbiol.

[12] Evans LR, Linker A (1973). Production and characterization of the slime polysaccharide of *Pseudomonas aeruginosa*. J Bacteriol.

[13] Mann EE, Wozniak DJ (2012). Pseudomonas biofilm matrix composition and niche biology. FEMS Microbiol Rev.

[14] Pritt B, O’Brien L, Winn W (2007). Mucoid *Pseudomonas* in cystic fibrosis. Am J Clin Pathol.

[15] Hentzer M, Teitzel GM, Balzer GJ, Heydorn A, Molin S (2001). Alginate overproduction affects *Pseudomonas aeruginosa* biofilm structure and function. J Bacteriol.

[16] Alkawash MA, Soothill JS, Schiller NL (2006). Alginate lyase enhances antibiotic killing of mucoid *Pseudomonas aeruginosa* in biofilms. APMIS.

[17] Charlesworth C, Saran V, Volpiana L, Woods H (2008). The role of biofilm structure in the mechanism of gentamicin and ciprofloxacin antibiotic resistance in *Pseudomonas aeruginosa* PAO1 biofilms. J Exp Microbiol Immunol.

[18] Simpson JA, Smith SE, Dean RT (1989). Scavenging by alginate of free radicals released by macrophages. Free Radic Biol Med.

[19] Pezzoni M, Lemos M, Pizarro RA, Costa CS (2022). UVA as environmental signal for alginate production in *Pseudomonas aeruginosa*: role of this polysaccharide in the protection of planktonic cells and biofilms against lethal UVA doses. Photochem Photobiol Sci.

[20] Wozniak DJ, Wyckoff TJO, Starkey M, Keyser R, Azadi P (2003). Alginate is not a significant component of the extracellular polysaccharide matrix of PA14 and PAO1 *Pseudomonas aeruginosa* biofilms. Proc Natl Acad Sci.

[21] Jennings LK, Storek KM, Ledvina HE, Coulon C, Marmont LS (2015). Pel is a cationic exopolysaccharide that cross-links extracellular DNA in the *Pseudomonas aeruginosa* biofilm matrix. Proc Natl Acad Sci.

[22] Le Mauff F, Razvi E, Reichhardt C, Sivarajah P, Parsek MR (2022). The Pel polysaccharide is predominantly composed of a dimeric repeat of α-1,4 linked galactosamine and N-acetylgalactosamine. Commun Biol.

[23] Friedman L, Kolter R (2004). Two genetic loci produce distinct carbohydrate-rich structural components of the *Pseudomonas aeruginosa* biofilm matrix. J Bacteriol.

[24] Colvin KM, Gordon VD, Murakami K, Borlee BR, Wozniak DJ (2011). The pel polysaccharide can serve a structural and protective role in the biofilm matrix of *Pseudomonas aeruginosa*. PLoS Pathog.

[25] Chew SC, Kundukad B, Seviour T, van der Maarel JRC, Yang L (2014). Dynamic remodeling of microbial biofilms by functionally distinct exopolysaccharides. mBio.

[26] Ma L, Conover M, Lu H, Parsek MR, Bayles K (2009). Assembly and development of the *Pseudomonas aeruginosa* biofilm matrix. PLoS Pathog.

[27] Byrd MS, Sadovskaya I, Vinogradov E, Lu H, Sprinkle AB (2009). Genetic and biochemical analyses of the *Pseudomonas aeruginosa* Psl exopolysaccharide reveal overlapping roles for polysaccharide synthesis enzymes in Psl and LPS production. Mol Microbiol.

[28] Murakami K, Ono T, Viducic D, Somiya Y, Kariyama R (2017). Role of *psl* genes in antibiotic tolerance of adherent *Pseudomonas aeruginosa*. Antimicrob Agents Chemother.

[29] Qin Z, Yang L, Qu D, Molin S, Tolker-Nielsen T (2009). Pseudomonas aeruginosa extracellular products inhibit staphylococcal growth, and disrupt established biofilms produced by *Staphylococcus epidermidis*. Microbiology.

[30] Serra R, Grande R, Butrico L, Rossi A, Settimio UF (2015). Chronic wound infections: the role of *Pseudomonas aeruginosa* and *Staphylococcus aureus*. Expert Rev Anti Infect Ther.

[31] Filkins LM, Graber JA, Olson DG, Dolben EL, Lynd LR (2015). Co culture of *Staphylococcus aureus* with *Pseudomonas aeruginosa* drives *S. aureus* towards fermentative metabolism and reduced viability in a cystic fibrosis model. J Bacteriol.

[32] Maliniak ML, Stecenko AA, McCarty NA (2016). A longitudinal analysis of chronic MRSA and *Pseudomonas aeruginosa* co-infection in cystic fibrosis: a single-center study. J Cyst Fibros.

[33] Alves PM, Al-Badi E, Withycombe C, Jones PM, Purdy KJ (2018). Interaction between *Staphylococcus aureus* and *Pseudomonas aeruginosa* is beneficial for colonisation and pathogenicity in a mixed biofilm. Pathog Dis.

[34] Billings N, Ramirez Millan M, Caldara M, Rusconi R, Tarasova Y (2013). The extracellular matrix component Psl provides fast-acting antibiotic defense in *Pseudomonas aeruginosa* biofilms. PLoS Pathog.

[35] Webb RB (1977). Lethal and mutagenic effects of near-ultraviolet radiation. Photochem Photobiol Rev.

[36] Fernández RO, Pizarro RA (1996). Lethal effect induced in *Pseudomonas aeruginosa* exposed to ultraviolet-A radiation. Photochem Photobiol.

[37] Fernández RO, Pizarro RA (1999). Pseudomonas aeruginosa UV-A-induced lethal effect: influence of salts, nutritional stress and pyocyanine. J Photochem Photobiol B.

[38] Chamberlain J, Moss SH (1987). Lipid peroxidation and other membrane damage produced in *Escherichia coli* K1060 by near-UV radiation and deuterium oxide. Photochem Photobiol.

[39] Bosshard F, Bucheli M, Meur Y, Egli T (2010). The respiratory chain is the cell’s Achilles’ heel during UVA inactivation in *Escherichia coli*. Microbiology.

[40] Hu ML, Tappel AL (1992). Potentiation of oxidative damage to proteins by ultraviolet-A and protection by antioxidants. Photochem Photobiol.

[41] Girard PM, Francesconi S, Pozzebon M, Graindorge D, Rochette P (2011). UVA-induced damage to DNA and proteins: direct *versus* indirect photochemical processes. J Phys: Conf Ser.

[42] Bäumler W, Regensburger J, Knak A, Felgenträger A, Maisch T (2012). UVA and endogenous photosensitizers--the detection of singlet oxygen by its luminescence. Photochem Photobiol Sci.

[43] Pezzoni M, Meichtry M, Pizarro RA, Costa CS (2015). Role of the Pseudomonas Quinolone Signal (PQS) in sensitising *Pseudomonas aeruginosa* to UVA radiation. J Photochem Photobiol B.

[44] Jagger J (1981). Near-UV radiation effects on microorganisms. Photochem Photobiol.

[45] Favre A, Hajnsdorf E, Thiam K, Caldeira de Araujo A (1985). Mutagenesis and growth delay induced in *Escherichia coli* by near-ultraviolet radiations. Biochimie.

[46] Ramabhadran TV, Jagger J (1976). Mechanism of growth delay induced in *Escherichia coli* by near ultraviolet radiation. Proc Natl Acad Sci.

[47] Pizarro RA (1995). UV-A oxidative damage modified by environmental conditions in *Escherichia coli*. Int J Radiat Biol.

[48] Pezzoni M, Tribelli PM, Pizarro RA, López NI, Costa CS (2016). Exposure to low UVA doses increases KatA and KatB catalase activities, and confers cross-protection against subsequent oxidative injuries in *Pseudomonas aeruginosa*. Microbiology.

[49] Pezzoni M, Pizarro RA, Costa CS (2018). Exposure to low doses of UVA increases biofilm formation in *Pseudomonas aeruginosa*. Biofouling.

[50] Pezzoni M, Pizarro RA, Costa CS (2020). Role of quorum sensing in UVA-induced biofilm formation in *Pseudomonas aeruginosa*. Microbiology.

[51] Pezzoni M, De Troch M, Pizarro RA, Costa CS (2022). Homeophasic adaptation in response to UVA radiation in *Pseudomonas aeruginosa*: changes of membrane fatty acid composition and induction of desA and desB expression. Photochem Photobiol.

[52] Hoerter JD, Arnold AA, Kuczynska DA, Shibuya A, Ward CS (2005). Effects of sublethal UVA irradiation on activity levels of oxidative defense enzymes and protein oxidation in *Escherichia coli*. J Photochem Photobiol B.

[53] Cadenas E, Ginsberg M, Rabe U, Sies H (1984). Evaluation of alpha-tocopherol antioxidant activity in microsomal lipid peroxidation as detected by low-level chemiluminescence. Biochem J.

[54] Rosenberg M, Gutnick D, Rosenberg E (1980). Adherence of bacteria to hydrocarbons: a simple method for measuring cell-surface hydrophobicity. FEMS Microbiol Lett.

[55] Rosenberg M (2006). Microbial adhesion to hydrocarbons: twenty-five years of doing MATH. FEMS Microbiol Lett.

[56] Khakimova M, Ahlgren HG, Harrison JJ, English AM, Nguyen D (2013). The stringent response controls catalases in *Pseudomonas aeruginosa* and is required for hydrogen peroxide and antibiotic tolerance. J Bacteriol.

[57] Hickman JW, Tifrea DF, Harwood CS (2005). A chemosensory system that regulates biofilm formation through modulation of cyclic diguanylate levels. Proc Natl Acad Sci.

[58] Rybtke MT, Borlee BR, Murakami K, Irie Y, Hentzer M (2012). Fluorescence-based reporter for gauging cyclic di-GMP levels in *Pseudomonas aeruginosa*. Appl Environ Microbiol.

[59] Tilbury RN, Quickenden TI (1988). Spectral and time dependence studies of the ultraweak bioluminiscence emitted by the bacterium *Escherichia coli*. Photochem Photobiol.

[60] Sakuragi Y, Kolter R (2007). Quorum-sensing regulation of the biofilm matrix genes (pel) of *Pseudomonas aeruginosa*. J Bacteriol.

[61] Ryder C, Byrd M, Wozniak DJ (2007). Role of polysaccharides in *Pseudomonas aeruginosa* biofilm development. Curr Opin Microbiol.

[62] Mikkelsen H, Sivaneson M, Filloux A (2011). Key two-component regulatory systems that control biofilm formation in *Pseudomonas aeruginosa*. Environ Microbiol.

[63] Tian L, Xu S, Hutchins WC, Yang C-H, Li J (2014). Impact of the exopolysaccharides Pel and Psl on the initial adhesion of *Pseudomonas aeruginosa* to sand. Biofouling.

[64] Rosenberg M, Barki M, Bar‐Ness R, Goldberg S, Doyle RJ (1991). Microbial adhesion to hydrocarbons (MATH). Biofouling.

[65] van Loosdrecht MC, Lyklema J, Norde W, Schraa G, Zehnder AJ (1987). The role of bacterial cell wall hydrophobicity in adhesion. Appl Environ Microbiol.

[66] Wang C, Jiang L, Wei D, Li Y, Sui X (2011). Effect of secondary structure determined by FTIR spectra on surface hydrophobicity of soybean protein isolate. Procedia Engineering.

[67] Sabra W, Lünsdorf H, Zeng AP (2003). Alterations in the formation of lipopolysaccharide and membrane vesicles on the surface of *Pseudomonas aeruginosa* PAO1 under oxygen stress conditions. Microbiology.

[68] Colvin KM, Irie Y, Tart CS, Urbano R, Whitney JC (2012). The Pel and Psl polysaccharides provide *Pseudomonas aeruginosa* structural redundancy within the biofilm matrix. Environ Microbiol.

[69] Chua SL, Ding Y, Liu Y, Cai Z, Zhou J (2016). Reactive oxygen species drive evolution of pro-biofilm variants in pathogens by modulating cyclic-di-GMP levels. Open Biol.

[70] Strempel N, Nusser M, Neidig A, Brenner-Weiss G, Overhage J (2017). The oxidative stress agent hypochlorite stimulates c-di-GMP synthesis and biofilm formation in *Pseudomonas aeruginosa*. Front Microbiol.

[71] Gao Q, Garcia-Pichel F (2011). Microbial ultraviolet sunscreens. Nat Rev Microbiol.

[72] Korgaonkar A, Trivedi U, Rumbaugh KP, Whiteley M (2013). Community surveillance enhances *Pseudomonas aeruginosa* virulence during polymicrobial infection. Proc Natl Acad Sci.

[73] Cardozo VF, Oliveira AG, Nishio EK, Perugini MRE, Andrade CGTJ (2013). Antibacterial activity of extracellular compounds produced by a Pseudomonas strain against methicillin-resistant *Staphylococcus aureus* (MRSA) strains. Ann Clin Microbiol Antimicrob.

[74] Park JH, Lee JH, Cho MH, Herzberg M, Lee J (2012). Acceleration of protease effect on *Staphylococcus aureus* biofilm dispersal. FEMS Microbiol Lett.

[75] HOLLOWAY BW (1955). Genetic recombination in *Pseudomonas aeruginosa*. J Gen Microbiol.

[76] Borlee BR, Goldman AD, Murakami K, Samudrala R, Wozniak DJ (2010). *Pseudomonas aeruginosa* uses a cyclic-di-GMP-regulated adhesin to reinforce the biofilm extracellular matrix. Mol Microbiol.

[77] Kirisits MJ, Prost L, Starkey M, Parsek MR (2005). Characterization of colony morphology variants isolated from *Pseudomonas aeruginosa* biofilms. Appl Environ Microbiol.

[78] Pawar SV, Messina M, Rinaldo S, Cutruzzolà F, Kaever V (2016). Novel genetic tools to tackle c-di-GMP-dependent signalling in *Pseudomonas aeruginosa*. J Appl Microbiol.

[79] Scilletta NA, Pezzoni M, Desimone MF, Soler-Illia GJAA, Catalano PN (2019). Transforming an inert nanopolymer into broad-spectrum bactericidal by superstructure tuning. Colloids Surf B Biointerfaces.

