# Supplementary material for: Role of Pel and Psl polysaccharides in the response of Pseudomonas aeruginosa to environmental challenges: oxidative stress agents (UVA, H2O2, sodium hypochlorite) and its competitor Staphylococcus aureus
Source: Microbiology (Reading). 2023 Feb 9;169(2):001301. doi: 10.1099/mic.0.001301 (PMC10197878; doi:10.1099/mic.0.001301)
Supplement: Supplementary material 1 [file mic-169-1301-s001.pdf]

Role of Pel and Psl polysaccharides in the response of *Pseudomonas aeruginosa* to environmental challenges: oxidative stress agents (UVA, H_2O_2, sodium hypochlorite) and its competitor *Staphylococcus aureus*

Romina Grossich*, Martin Lemos Vilches *, Cristina S. Costa, Magdalena Pezzoni**

Supplementary information

Fig. S1

Strain	A	B1	B2	B3
PAO1 control	-1.29165	0.01135	$4.90842E^{-5}$	$8.01166E^{-8}$
PAO1 UVA	-1.30523	0.0091	$3.87818E^{-5}$	$6.75189E^{-8}$
pel control	-1.31235	0.00729	$1.96075E^{-5}$	$2.14391E^{-8}$
pel UVA	-1.33684	0.00446	$1.60158E^{-7}$	$1.379E^{-8}$
psl control	-1.30984	0.0092	$3.54559E^{-5}$	$5.71568E^{-8}$
psl UVA	-1.36111	0.00628	$1.24136E^{-5}$	$1.40341E^{-8}$
pel psl control	-1.21682	0.00946	$4.8836E^{-5}$	$8.87966E^{-8}$
pel psl UVA	-1.31102	0.00565	$1.26979E^{-5}$	$1.19839E^{-8}$
wspF control	-1.27217	0.00483	$9.20196E^{-6}$	$4.37424E^{-8}$
wspF UVA	-1.26941	0.00264	$2.32956E^{-5}$	$6.95391E^{-8}$

Parameters of the polynomial regressions ($Y= A + B1.X + B2.X^2 + B3.X^3$) of the fitted growth curves of PAO1, *pel*, *psl*, *pelpsl* and *wspF*strains under sublethal UVA exposure or in the dark (control).

Fig. S2

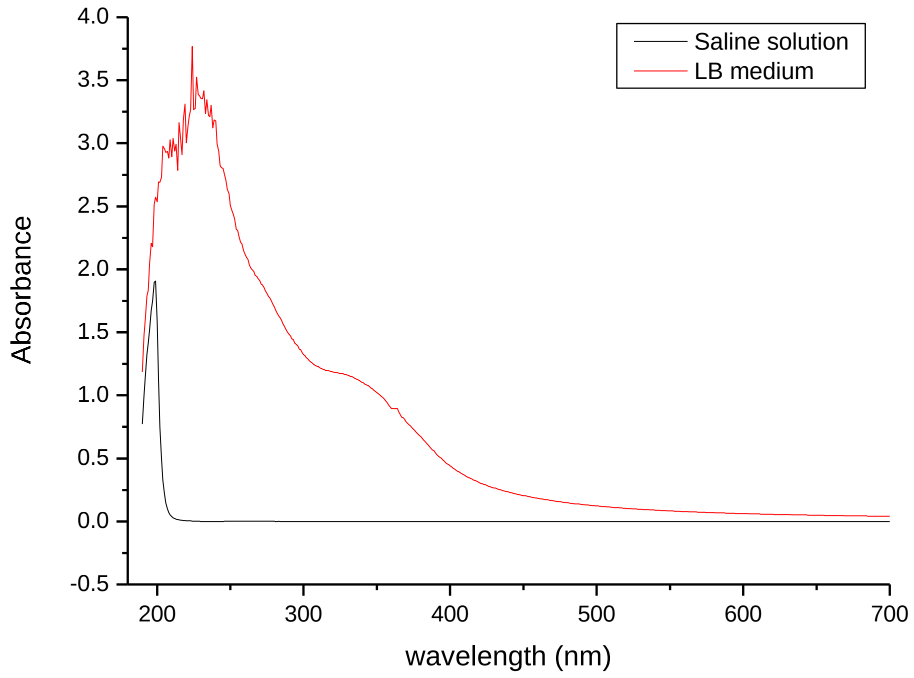

Representative UV-Vis spectrum of LB or saline solution (UVA range: 320-400 nm)

Fig. S3

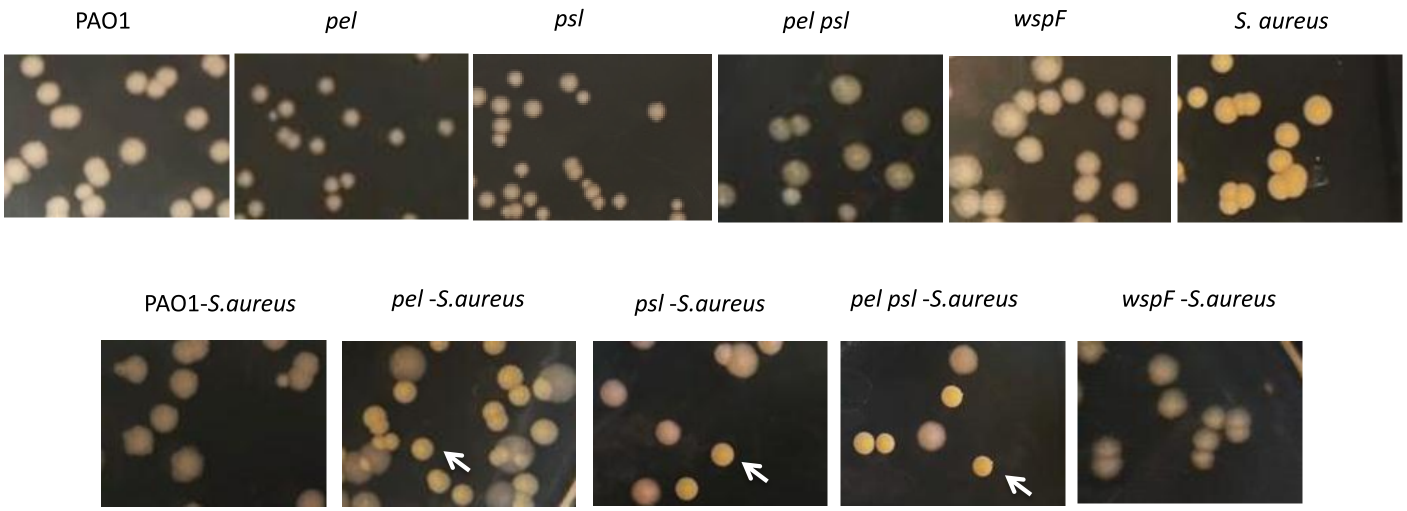

Representative photographs showing the colony morphology of all the strains used in this study. The arrows point to *S. aureus* colonies in mixed cultures.
